# Islet Encapsulation: New Developments for the Treatment of Type 1 Diabetes

**DOI:** 10.3389/fimmu.2022.869984

**Published:** 2022-04-14

**Authors:** Qi Zhang, Carmen Gonelle-Gispert, Yanjiao Li, Zhen Geng, Sandrine Gerber-Lemaire, Yi Wang, Leo Buhler

**Affiliations:** ^1^ School of Medicine, University of Electronic Science and Technology of China, Chengdu, China; ^2^ Faculty of Science and Medicine, University of Fribourg, Fribourg, Switzerland; ^3^ Clinical Immunology Translational Medicine Key Laboratory of Sichuan Province, Center of Organ Transplantation, Sichuan Academy of Medical Science and Sichuan Provincial People’s Hospital, Chengdu, China; ^4^ Group for Functionalized Biomaterials, Institute of Chemical Sciences and Engineering, Ecole Polytechnique Fédérale de Lausanne (EPFL), EPFL SB ISIC SCI-SB-SG, Lausanne, Switzerland; ^5^ Institute of Organ Transplantation, Sichuan Provincial People's Hospital, University of Electronic Science and Technology of China, Chinese Academy of Sciences, Sichuan Translational Medicine Research Hospital, Chengdu, China

**Keywords:** islet, material, immunogenicity, encapsulation, transplantation

## Abstract

Islet transplantation is a promising approach for the treatment of type 1 diabetes (T1D). Currently, clinical islet transplantation is limited by allo - and autoimmunity that may cause partial or complete loss of islet function within a short period of time, and long-term immunosuppression is required to prevent rejection. Encapsulation into semipermeable biomaterials provides a strategy that allows nutrients, oxygen and secreted hormones to diffuse through the membrane while blocking immune cells and the like out of the capsule, allowing long-term graft survival and avoiding long-term use of immunosuppression. In recent years, a variety of engineering strategies have been developed to improve the composition and properties of encapsulation materials and to explore the clinical practicality of islet cell transplantation from different sources. In particular, the encapsulation of porcine islet and the co-encapsulation of islet cells with other by-standing cells or active ingredients for promoting long-term functionality, attracted significant research efforts. Hydrogels have been widely used for cell encapsulation as well as other therapeutic applications including tissue engineering, cell carriers or drug delivery. Here, we review the current status of various hydrogel biomaterials, natural and synthetic, with particular focus on islet transplantation applications. Natural hydrophilic polymers include polysaccharides (starch, cellulose, alginic acid, hyaluronic acid, chitosan) and peptides (collagen, poly-L-lysine, poly-L-glutamic acid). Synthetic hydrophilic polymers include alcohol, acrylic acid and their derivatives [poly (acrylic acid), poly (methacrylic acid), poly(acrylamide)]. By understanding the advantages and disadvantages of materials from different sources and types, appropriate materials and encapsuling methods can be designed and selected as needed to improve the efficacy and duration of islet. Islet capsule transplantation is emerging as a promising future treatment for T1D.

## Introduction

Diabetes mellitus (DM) describes a group of metabolic disorders characterized by high blood glucose levels, and patients with DM have an increased risk of developing a number of serious life-threatening pathologies like retinopathy and cardiovascular diseases (1), resulting in higher medical care costs, reduced quality of life and increased mortality (2). It was estimated that in 2017, there were 451 million (age 18–99 years) people with diabetes worldwide. These figures were expected to increase to 693 million by 2045 (3). Patients with type 1 diabetes (T1D) depend on exogenous insulin supply, however long-term clinical insights have shown the failure of insulin preparations to fully replicate biological actions of endogenous insulin (4). For patients suffering from severe and repeated hypoglycemia events, islet transplantation demonstrated beneficial effects.

Unfortunately, islet allo or xenotransplantation is limited by inflammatory and immune reactions resulting in low survival (5, 6). Transplanted islets are initially destroyed by instant blood-mediated inflammatory reaction (IBMIR) during intraportal infusion of allogeneic or xenogeneic islets (7). Simultaneously, cell transplantation by intraportal infusion may cause bleeding, thrombosis and other related complications (8). The coagulopathy may be due to the release of tissue factor (TF), a physiological trigger for clotting that is secreted by islet cells (9).

Shortly after transplantation, acute immune responses are initiated as chemokines and chemokine receptors induce the recruitment and activation of various leukocytes (10). Activated immune cells cause damage to the islets by releasing pro-inflammatory cytokines and reactive oxygen species (ROS) (11).

Currently, prolonged graft survival is achieved by using continuous immunosuppressive drugs. However, the toxicity associated with long-term immunosuppression includes various adverse effects, such as opportunistic infections, nephrotoxicity, myelosuppression and cancer (12). Immunosuppressive medication is also detrimental to the survival and functionality of the transplanted cells. These severe side effects may significantly reduce the benefit of islet transplantation. Moreover, long-term use of immunosuppressive agents can also significantly impair glucose tolerance and lead to increased insulin resistance (13).

**Figure 1:**
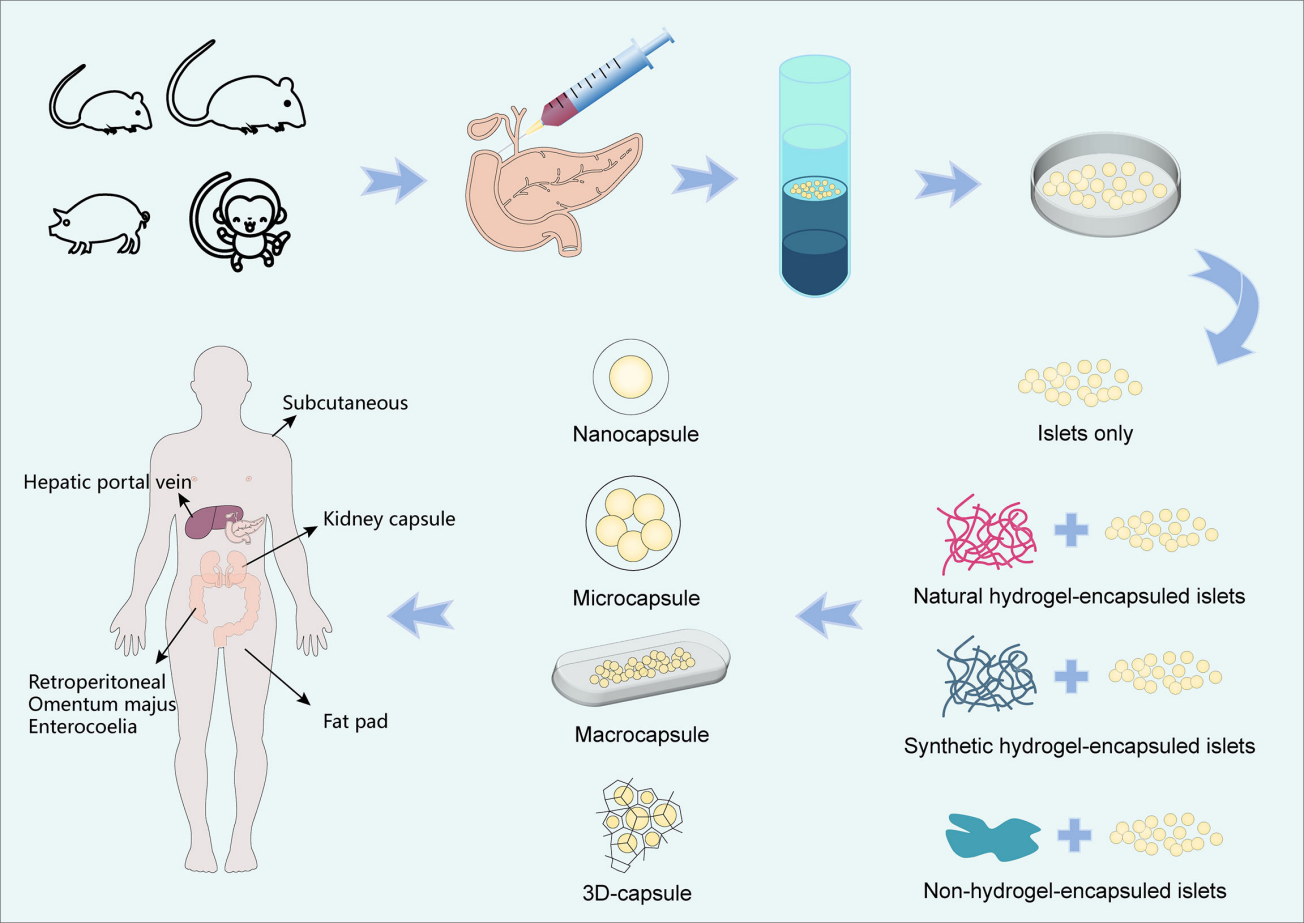
Flow chart of islet encapsulation and transplantation. There are many sources of islet cells, such as mice, rats, porcines and monkeys, etc. We injected collagenase into pancreas and isolated islets by density gradient centrifugation after pancreatic filling. The islets are capsuled with natural hydrogels, synthetic hydrogels and other types of hydrogels, forming capsules of different sizes (Nanocapsule, Microcapsule, Macrocapsule and 3D-capsule) and then transplanted. Common sites of transplantation are subcutaneous, kidney capsule, hepatic portal vein, retroperitoneal, omentum majus, enterocoelia, fat pad, etc.

**Figure 2:**
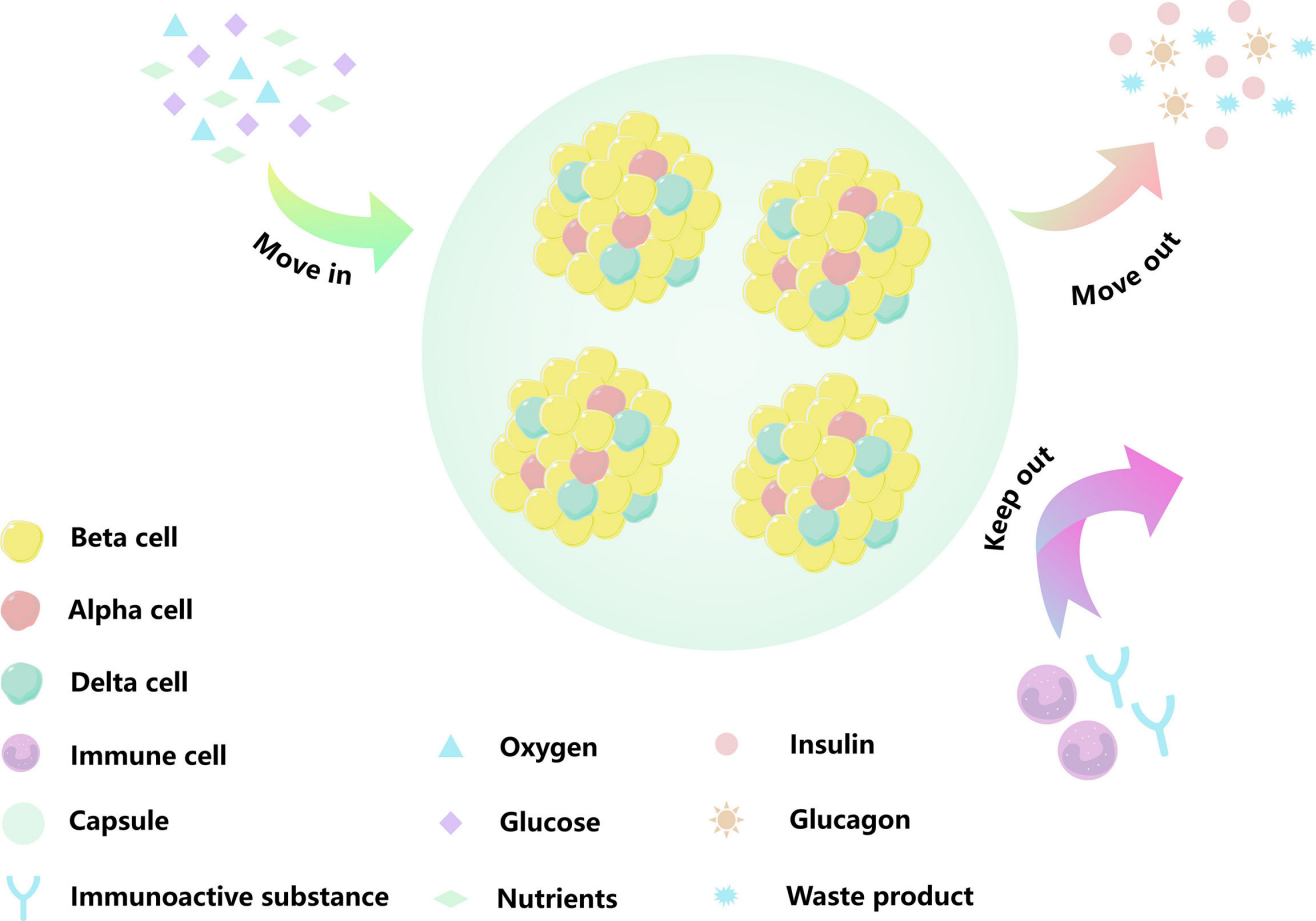
Schematic diagram of an encapsulated islet. Biomaterials can provide shelter for islet cells, minimize the immune response, and mimic a process by which material moves in and out of cell. The yellow cells are beta cells, the red cells are alpha cells, the green cells are Delta cells, the purple cells are immune cells, the green circular background is capsule, the blue Y-shape is immune active substance. The blue triangle is oxygen, the purple square is glucose, the green diamond is nutrient, the pink circle is insulin, the orange sun is glucagon, and the blue star is metabolic waste.

**Figure 3:**
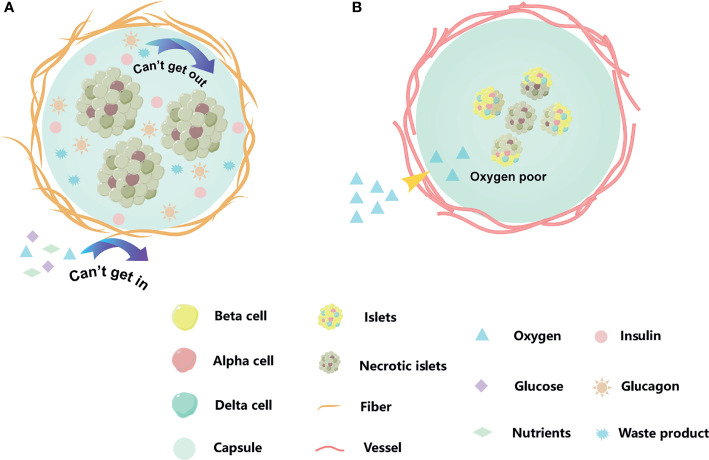
Existed problems for islets encapsulation. **(A)** A dense peri-capsular fibrotic overgrowth, FBR usually limits the diffusion of nutrients and oxygen to the implant, or prevents insulin release and waste discharge. **(B)** The encapsulation we moved into recipient acted as a physical barrier, blocking blood vessels from growing into the capsule. The cell of capsule center by hypoxia. The orange strips are fiber, and the red cords are blood vessel. When islet cells are affected by hypoxia or cellular metabolites, they change from bright yellow to dark gray.

To prevent immune rejection and avoid continuous immunosuppression, cell encapsulation technology is a promising alternative relying on the immobilization of endocrine cells into semi-permeable hydrogel matrices which protect them from the immune system (**Figure 1**). Currently, there are several islet encapsule approaches. The main strategy consists in the use of polymeric hydrogel microcapsules which protect islets from contact and attack by immune cells. Their specific pore size makes the biomaterial membrane permeable to small nutrients and secreted insulin, but prevents the diffusion of immune cells and large molecules such immunoglobulin into the capsule core (**Figure 2**) (14). A complementary strategy is to co-embed islets with by standing cells, molecular cargos and biomolecules that promote islet survival and function. Nevertheless, islet immobilization into hydrogel microcapsules result in the exacerbation of the hypoxic situation (**Figure 3**) (15). Such detrimental effect can be addressed by the use of macrocapsule devices which promote angiogenesis and allow for extracorporeal oxygen supply. Although some researchers summarized the islet encapsulation, they were focusing on different aspects, such as the size of the encapsulated material, immune fusion (16, 17), the immune mechanisms of islet transplantation (18), clinical trials and their influencing factors (19) or the encapsulation of stem cell-derived islets (sc-islets) (20). This review focused on the classification of the islet encapsulation materials, highlighting their application in islet transplantation. In order to better understand the materials, we summarized the designing of the encapsulation materials, the generation of the fibers, and also the related *in vivo* studies.

## Design Rules for Islets Encapsulation

For designing islet encapsulation strategies, a series of parameters needs to be carefully assessed, including the content of the graft, the expected capsule size, the targeted mode of surgical operation, the implantation site, donor-recipient differences, environmental conditions within the organism, etc. The selection and development of the appropriate hydrogel is key to the success of the transplantation. We discuss the various aspects that determine optimal hydrogels selection and design in conjunction with the structure-property-function of stimulating-type polymers (21).

### Biocompatibility of Encapsulated Materials

T1D is a complex immune disease. Therefore, biocompatibility is the most important feature in the selection of islet encapsulation materials in order to avoid any deleterious immune response from the recipient. Biomaterial properties such as topography, surface charge and mechanical stability can also be modified to modulate the biocompatibility of the encapsulation matrix toward both the islet content and the host, thus influencing cell survival and recipient tolerance to the implanted material.

Topographical features (e.g., geometric arrangement) may play a role in guiding cell behavior (e.g., adhesion, migration). It has been found that the pore size of the encapsulated material affecting the elongation and transition of macrophages (M1 to M2 phenotype) (22), achieving functional regeneration of tissues (23). Porous matrix can be prepared by various methods such as fiber bonding, salt impregnation, foaming, three dimensional (3D) printing, and freeze-drying (24).

Charge is the determinant of the aqueous environment inside the hydrogel (25). By controlling the hydrogel internal water structure, the solute diffusion rate in the hydrogel can be changed. When the hydrogel has a neutral charged structure, it strongly binds water molecules and effectively inhibits the adhesion of bioactive substances (26). Chemical modification of zwitterionic polymers or charge neutralization of hydrogels with PEI and melanin can achieve better immune-shielding performance (27).

The mechanical stability of hydrogels affects the ability of encapsulated cells to maintain long-term stability *in vivo*. In this case, the mechanical properties of the hydrogel can be regulated by covalent cross-linking, grafting or mixing with other suitable polymer chains to prevent instability under physiological conditions. On the other hand, the stiffness of the hydrogel affects the strength of the foreign body reaction (FBR) it induces *in vivo* (28). It still remains a challenge to produce hydrogels which reach a balance between adequate cell protection to maintain their long-term functionality while causing as little FBR as possible through proper regulation.

### pH (*Pondus hydrogenii*)

The pH value of human body fluid ranges from 7.35 to 7.45. A decrease of the pH value by 0.1 results in a decrease of insulin activity by 30%. Therefore, the pH value should be carefully taken into account when designing encapsulation hydrogels. pH affects the properties of hydrogel materials, usually sensitive hydrogels containing anion and cation groups such as -COO-, -NH3+, -OPO3+ accept or give ions through pH changes to achieve gel-solution changes. These changes are mainly attributed to electrostatic repulsion and osmotic forces within the backbone chain (29). The same materials were prepared in solutions with different pH values, and the change in pH value led to a significant change in the interface-cell interactions (30).

### Enzyme

In recent years, bioactive hydrogels have been generated by non-covalent co-assembly with enzymes. The different structures and functions of hydrogels are achieved through *in situ* enzyme dynamic cross-linking, enzymatic polymerization and interfacial enzyme assembly effects to build an efficient interoperative responsive microenvironment (31). By scavenging excess enzymes as catalase (CAT) and superoxide dismutase (SOD) in the hydrogel, the islets could have prolonged viability by eliminating reactive oxygen species (ROS) (32). Additionally, there are also metal-containing nano-enzymes, which mimic natural enzyme-like activities with smaller size, enhanced stability and lower price,. However, it is important to note that enzymes, may cause damage to the islet microenvironment and ultimately lead to a poor prognosis even at the initial stage of transplantation.

### Temperature

Current islet transplantation still relies mainly on isolation and transplantation from deceased donors. In case of mismatch between donor and recipient time, there is a need to find efficient cryopreservation techniques. Combining cold-sensitive hydrogels with appropriate encapsulation techniques allows islets to not only survive the freeze-thaw process, but also provide a natural barrier to islets *in vivo (* 33). In a specific temperature range, thermo-responsive polymers undergo phase transition due to the formation of intermolecular hydrogen bonds, hydrophobic interactions and physical entanglement of polymer chains (34). Conjugation a hyaluronic acid with different sulfation degrees and an amine-terminated poly(N-isopropylacrylamide) resulted in a thermogel that was not only highly compatible with rabbit corneal cells, but its degree of sulfation also had a lasting anti-inflammatory effect (35).

### Hydrogel Preparation Methods

Large-Scale manufacturing includes preparing injectable and pre-synthesized hydrogels. Injectable hydrogels enable islet transplantation by injection at the implantation site due to their fluidic nature. The advantages of injectable hydrogels over pre-synthesized solid hydrogels are their non-invasive nature and adaptability to the host tissue space (36). Most injectable hydrogels do not maintain the structural integrity required to protect cells after injection. There can also be gelation during the injection, clogging the pillow and preventing the transplant process (37). When designing injectable hydrogels, attention should be paid to several aspects (36). The first is the shear thinning, which cushions the damage to the fragile cells from shear forces during injection. The second is thixotropy, which determines the rate at which the injectable hydrogel transforms into a robust protective capsule. The means of triggering also has to be considered, which will determine the suitability of the hydrogel. Injectable hydrogel can be prepared by non-toxic chemical cross-linkers, enzymatic cross-linkers, physical interactions, etc. Alginate has natural, biocompatible and economical properties. However, its potential as an injectable hydrogel is limited by poor control of gelation. Alginate based hydrogels were prepared by ionic cross-linking methods. The physicochemical properties of injectable alginate such as gel formation time, hardness and porosity can be adjusted using different concentrations of Na_2_HPO_4_ (38). In addition to different concentrations of ions, variation on the ion sources can also modulate the alginate gelation process, resulting in a different range of physicochemical properties. The human body environment and circulating biomolecules were also exploited for the development of injectable hydrogels. The injectable hydrogel obtained by mixing plasma with the hydroxypropyl methylcellulose (HPMC) serves as the base environment for islet encapsulation. When this liquid is injected into the body, it rapidly polymerizes into a fibrin gel after being influenced by circulating thrombin *in vivo*. The nest-like structure formed by the polymerization provides protection to the islets and improves their survival rate (39).

Solid hydrogels, also known as pre-synthesized hydrogels, pre-immerse islets *in vitro*, and the incorporation of extracellular matrix (ECM) components greatly enhance islet functions. Solid hydrogels provide good encapsulation conditions for islet cells to be implanted, but may be accompanied by various types of surgical risks during the transplantation process. At the same time, the already fixed shape is a considerable challenge in adapting to the host’s tissue space and mechanical properties, which are necessary to establish host-implantation interactions (40). Prefabricated hydrogels can be prepared from a wide variety of materials, involving various engineering methods and potential combination with biological factors and active substances. These aspects are not detailed in the present review.

## Hydrogels as Biomaterials for Cell Encapsulation

Hydrogels are composed of cross-linked macromolecules that form 3D structures with high water content. Cross-linking strategies include physical interactions (hydrogen bonding), ionic interactions and chemical conjugation which ensure the stability and physical integrity of hydrogels in aqueous medium (41). In addition, the cross-linked network provides the hydrogels with tunable mechanical properties (strength and elasticity) and determines their diffusion properties and internal transport capacity as encapsulation material (42). The polymer network maintains the shape and volume of hydrogels by balancing capillary, osmotic and hydration forces to protect cells (43). At the same time, their high-water content mimics the softness of natural tissues while allowing the bi-directional diffusion of nutrients, metabolites and wastes (44, 45). These characteristics make hydrogel ideal cell encapsulation materials. Most water-soluble or hydrophilic polymers can become hydrogels upon chemical or physical cross-linking conditions. However, the morphology and properties of the final capsules highly depend on the composition of the polymeric materials and the technology applied for capsule formation. A large variety of natural and synthetic materials were investigated in the context of cell encapsulation (**Table 1**).

**Table 1:** Hydrogels as biomaterials for cell encapsulation.

Material	Honor	Recipient	Graft	Site	Result	Reference
Alginate	Wistar rats	Wistar rats	2,000~3,000 islets	intraperitoneal	normoglycemia (3 wks)	(14)
Collagen	C57BL/6J or CD1 mice	NOD.CB17-Prkdc^scid^/J or C57BL/6J	500 islets	subcutaneous	normoglycemia for 14d (immunocompromised), 90d (syngeneic), and 40d (allogeneic).	(46)
Silk	BALB/c or C57Bl/6		islets, ECM and MSCs		islets remained viable and SI was 4.4(7d)	(47)
Alginate&gelatin	double heterozygous crossbreed mice	NSG mice	500 islets	subcutaneous	detect GFP expression and image islets(7d)	(85)
Alginate-poly-L-lysine	Rats	Mice	1000 islets and 1mg MSC-CellSaic	the skin and muscles of the abdomen	a large number of vessels normoglycemia (by 1 month)	(49)
Alginate-polylysine-alginate	Porcine	Monkeys	3-7 x 10^4^ islets at a time	intraperitoneal	normoglycemia (120d~804d)	(50)
Alginate&Teflon	German landrace sows	Gottingen Minipigs	3000 IEQ	subcutaneous	no signs of local inflammatory or fibrotic reactions(13d), the Glucose stimulated insulin release (GSIR) is 6.7	(51)
PEG-RGD	Balb/c	B6	1,200 IEQ	Epididymal fat pad (EFP)	normoglycemia (>100d)	(52)
PEG-RGD	B6(GFP)	NOD mice	800 IEQ	gonadal adipose tissue	high density of GFP signal and significant vascularization (9 wks)	(52)
PEG-MAL-RGD	Rat	Rat	1,500 islets	mesentery	new vessels (by 1 wk)	(53)
Biotin-PEGNHS/SA/biotin-PEG-GLP-1	Rat	_	islets	_	increased insulin secretion	(54)
PEG-b-PLA	Piglets	C57/BL6	neonatal porcine islet-like cell clusters (NPCCs)	kidney capsule	little infiltration of immune cells	(55)
PEG-DA	Porcine	Athymic mouse	islets	kidney capsule	no fibrosis (>2 wks) viable porcine islet(>100d)	(56)
Triazole-zwitterionic (TR-ZW)	Sprague-Dawley rats	C57BL/6	500 islets	subcutaneous	normoglycemia (1 month)	(57)
PEG-inhibitory peptide	_	_	Mouse insulinoma (MIN6) cells	_	reduce the death of MIN6 cells	(58)
PLG	BALB/c	C57BL/6	1,000-1,100 islets	epididymal fat pad (EFP)	normoglycemia (200d)	(59)
PLG-Treg	NSG mice	NOD mice	300 islets+3x10^6^Treg	intra-abdominal fat	normoglycemia (>99d)	(60)

Hydrogels derive from natural or synthetic polymeric materials (61). Natural hydrophilic polymers include polysaccharides (starch, cellulose, alginic acid, hyaluronic acid, chitosan, etc.) and peptides (collagen, poly-L-lysine, poly-L-glutamic acid), etc.). Synthetic hydrophilic polymers include poly (vinyl alcohol), acrylic acid and their derivatives (poly (acrylic acid), poly (methacrylic acid), poly(acrylamide), etc.). In recent decades, hydrogels have been widely used in wound dressings (62), tissue engineering (63), cell carriers (64), drug delivery (65), antifouling coatings (66).

## Hydrogel Derived From Natural Polymers

Hydrogels produced from natural materials mostly derive from polysaccharides such as starch, cellulose, alginate and collagen, as well as peptides such as elastin and poly-L-lysine (67).

Alginate is a polysaccharide mainly found in the cell wall and intercellular mucilage of brown algae, but also in some bacteria of *Azotobacter* sp. and *Pseudomonas* sp. It is commonly applied as cell microencapsulation material due to its low toxicity, low immune response, and ability for instantaneous formation of ionic hydrogel in the presence of divalent cations such as Ca^2+^ and Ba^2+^ (68, 69). Alginate is a linear copolymer composed of 1-4-linked β-D-mannuronate (M) and (or) α-L-guluronate (G) residues. The physical properties of alginate molecules are determined by the ratio and distribution of the three types of blocks: MG-blocks, MM-blocks, GG-blocks (70). The fast gelation properties of alginate upon contact with a solution containing divalent cations allow for cell microencapsulation under mild conditions (68). Several parameters, such as the association constant, the ionic strength and the affinity for the different blocks, depends on the nature of the gelling cations. For instance, Ba^2+^ preferably associates with GG and MM blocks while Ca^2+^ favors ionic interactions with GG and MG blocks. These different association patterns have significant impact on the porosity, rigidity, elasticity and mechanical resistance of the hydrogel (71). The induction of immune responses in organisms by alginate is mainly related to its composition. Several studies highlighted that M-blocks and MG-blocks, but not the G-blocks, stimulated the cytokine production (72). There are claims that immunogenicity is related to the purity of the alginate, and that mannuronic acid-rich alginate is usually less viscous, allowing the gel to have a higher alginate content. Implantation of these purified alginate capsules into mouse models with elevated macrophage activity also showed no FBR (73).

Collagen is a fibrous protein and displays at least one triple-helical domain. It is an important component of the animal ECM and ensures the structural integrity of tissues and organs (74). The mechanical properties and stability of collagen are lower than the natural state due to the disruption of the assembly structure and natural cross-linking during the extraction process, which requires the induction of exogenous cross-linking for its optimization (75). Cross-linking may lead to collagen denaturation and exposure of antigenic determinants clusters to induce immune responses (75). Moreover, due to the differences in species, natural collagen may cause immune problems such as allergies as well as disease propagation.

Unlike previous collagen formulations used for islet encapsulation, Clarissa et al. (46) used oligomeric collagen to encapsulate islets, which retains natural intermolecular cross-linking and is able to rapidly self-assemble into highly interconnected D-band fibrous scaffolds upon neutralization (76). These structures were applied to the encapsuling of mouse islets. The highest functionality of the embedded cells, measured *in vitro* over 14 days, was achieved at a concentration of oligomer of 3 mg/mL. The ability of these systems to maintain normoglycemia after subcutaneous injection in the back was demonstrated *in vivo* with different models: 14 days in NOD.CB17- Prkdc^scid^/J mice, 90 days in syngeneic mice and 40 days in allogeneic mice. Islet microorganisms interact with extracellular matrix components such as type I collagen fibers to maintain islet function and homeostasis. The high biocompatibility of type I collagen *in vivo* supports small molecule transport while reducing infiltration and activation of inflammatory cells, providing a new solution for subcutaneous islet transplantation.

Silk comes from a wide range of sources, such as silkworm, spider silk, and peanut silk, which results in differences in the composition of silk protein. Treated silk proteins have low antigenicity and rarely cause immune reactions when implanted *in vivo* (77). The main factors causing an immune response are the immune cells and signaling molecules adsorbed on the material (78). The performance of islets encapsulated in silk materials was significantly enhanced by co-encapsulation with fibroin, a protein presenting strong mechanical properties and low immunogenicity (79, 80). Davis N et al. (47) demonstrated that the co-encapsulation with MSCs resulted in a 2.3 fold increase of the stimulation index (SI) and that additional co-encapsulation of fibroin led to 4.4 fold SI enhancement, as compared with pure silk encapsulated islets.

## Combination of Polysaccharide and Polypeptide Polymers

**Figure 4:**
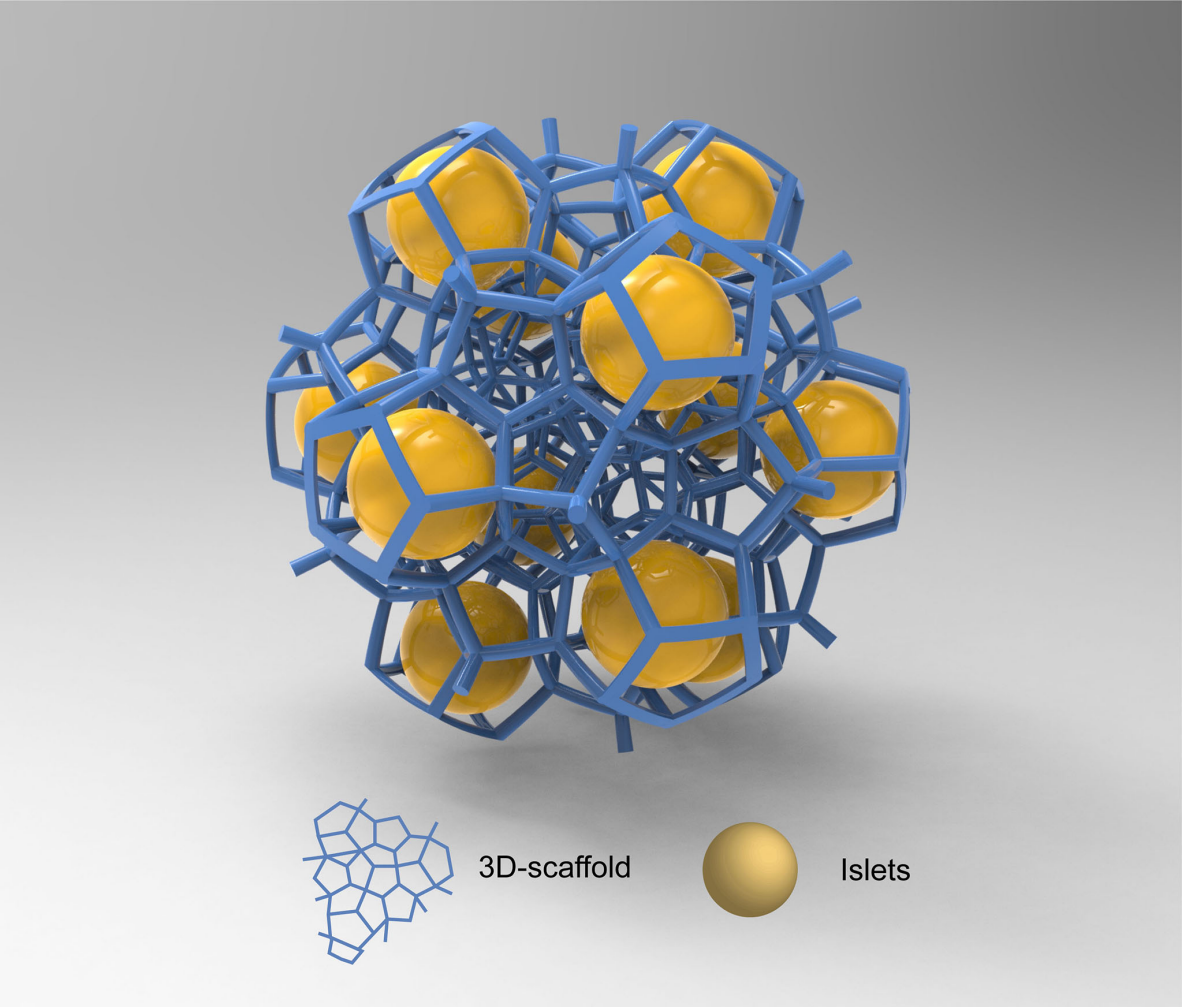
3D-capsule schematic diagram. Special biomaterials are used as inks to print 3d scaffolds in which cells are housed. The yellow spheres in the figure are islet cells, and the blue scaffold is 3D scaffold.

The 3D-printed capsule technology uses polymers as inks and piezoelectric or thermally driven mechanisms to process high-resolution bio-ink droplets (**Figure 4**) (81). The precise spatial control achieved with 3D-printing allows for the immobilization of cells into well-defined microstructures such as cylindrical filaments (82). The variety of complex structures produced through this technology has the potential to overcome the technical difficulties faced by conventional islet encapsulation. In particular, conventional polymeric hydrogel microcapsules generally do not support the formation of a vascular network after transplantation, thus impairing the efficient delivery of oxygen and nutrients to the entrapped cells (83). A few studies investigated the ability of 3D printed scaffolds to promote the formation of a vascular network around encapsulated islets (84). Marchioli et al. produced 3D-printed hydrogels composed of 4% alginate and 5% gelatin (85). By controlling the shape and porosity of the scaffolds, they observed the formation of blood vessels toward the device and promotion of angiogenesis. Compared with conventional spherical hydrogels, 3D-printed scaffolds prevented islets from aggregation, thereby increasing the surface area for small molecule exchange.

Currently, 3D bio-printing technology is still at an early stage of development, and its application is still subject to many limitations. The relatively weak mechanical properties of bio-inks used in 3D printing does not allow for the formation of hydrogels requiring high mechanical strength (86). The bio-inks used in 3D printing should present low viscosity (<10 mPa/s) to avoid nozzle blockage, thus limiting its applicability (87, 88). Further research is needed to develop hydrogels with sufficient viscosity and mechanical properties to suit the plotting function and islets functionality. At the same time, the incorporation of ECM components, endothelial cells and vascular endothelial growth factor (VEGF) into the bio-ink can make the printed model more similar to the living environment of islet cells (89), thus enhancing their biological function. Otherwise, 3D printing technology can achieve fast manufacturing throughput and maintain high cell vitality. Overall, 3D printing is seen as one of the most promising encapsulation approaches because it can produce clinically relevant multi-component devices in a short period of time.

## Co-Encapsulation Strategies Based on Natural Polymer Derived Hydrogels

Mesenchymal Stroma Cells (MSCs) reduce the immune response by releasing cytokines and growth factors (90, 91) and also have the potential to induce angiogenesis and repair of damaged tissues (92). MSC-CellSaic is a cell transplantation platform consisting of MSCs and recombinant peptides (RCP) arranged in a mosaic shape to avoid cell death (93). Ryo Kogawa et al. used CellSaic technology in a two-stage implantation protocol. Fist, an empty mesh bag was placed into the abdominal cavity of Balb/C mice, which could induce the formation of blood vessels around and in the band. Subsequently, alginate microencapsuled rat islets coated with poly-l-lysine (PLL) and MSC-CellSaic were inserted into the nylon mesh bag. Glucose levels were significantly reduced in the mice treated with the combination of islets and MSC-CellSaic as compared with the control group without MSCs (49).

Using alginate-poly-L-lysine-alginate microcapsules, porcine islets were transplanted into diabetic Cynomolgus monkeys. Seven of the nine monkeys achieved normal fasting blood glucose with insulin independence for periods ranging from 120 days to 804 days (50). As mentioned earlier in relation to alginate immunogenicity, mannuronic acid residues act as cytokine inducers causing fibrosis in implanted microcapsules. When alginate microcapsules are covered with PLL, the mechanical stability and permeability of the encapsulation material is improved.

Considering the severe hypoxia and immediate blood-mediated inflammation that portal vein and intraperitoneal transplants face, extravascular islet transplantation has been explored. A device, termed ‘β Air’ device, consists of islets immobilized in alginate and a hydrophilic Teflon membrane impregnated with alginate. The alginate and membrane barrier protect the islets from contact with immune cells, complements and antibodies, while a subcutaneous port supplies oxygen from the outside. Under these conditions, allogeneic pig islets were explanted after 13 days and the islet function remained active. The transplanted pocket was clean and showed no signs of local inflammation or fibrotic reaction, providing a new strategy for the feasibility of pig islet cell transplantation (51). After testing the device in small and large animal models, the method was applied to a patient with long-term T1D. The chamber with allogeneic islet was placed in a preperitoneal pocket and the oxygen supplying device was implanted close to the incision. The allogeneic islets survived and were able to maintain insulin secretion in response to the variation of blood glucose level for 10 months. A thin fibrous capsule appeared at the implantation site without any signs of inflammation (94).

## Artificial Hydrogels

While hydrogels deriving from natural polymers display adequate biocompatibility and low production cost, their stability under physiological conditions is often limited. In contrast, the production of artificial hydrogels, also called synthetic hydrogels, can provide improved control over the material properties, including pore size, mechanical strength and elasticity. Synthetic hydrogels have higher mechanical resistance, extended durability and wider application range as compared with natural hydrogels (41).

Poly (ethylene glycol) (PEG) hydrogels have been widely used in implantable devices due to their advantages such as convenient and rapid preparation. However, PEG is less biocompatible than natural polysaccharides and does not fully protect encapsulated cells from cytokines attacks (95). The combination of PEG with controlled amounts of components of the native islet microenvironment led to promising cell transplantation devices (96). The proteins naturally associated with the islet basement membrane (fibronectin, laminin, type IV collagen) were co-encapsulated with mice islets and resulted in enhanced *in vitro* insulin secretion up to 7 days post-encapsulation (97–99). The effect was further reinforced by the incorporation of bone marrow derived MSCs within the hydrogel matrix (48).

Weber et al. combined murine islets with type IV collagen and laminin in three-dimensional PEG hydrogels resulting in a two-fold and four-fold increase in insulin secretion, respectively, compared with islets that were not encapsulated with ECM proteins. Hydrogel containing both matrix proteins and more than 75% laminin produced about six times higher insulin secretion when compared with the islets encapsulated in the absence of matrix proteins (98).

Arginine-glycine-aspartic acid (RGD) is present in several ECM proteins, such as fibronectin, collagen type I, allowing for cell adhesion and its modified substance improves biocompatibility (100). Recent studies have shown that the incorporation of oligo-peptide RGD into PEG hydrogels reduces FBR (101). Weaver et al. have developed a microfluidic system, that produces smaller diameter microgels (310 + 14 μm) based on synthetic PEG, allowing their implantation within a vascularized retrievable site. Under these conditions, murine islets were encapsulated in PEG-RGD hydrogels and showed insulin secretion capacity up to 100 days (52). Using this system, green fluorescent protein-expressing (GFP-expressing) islets were transplanted into non-obesity diabetes (NOD) mice. During the 9-week period of follow-up, high density GFP signal was detected in recipient mice and significant angiogenesis was observed within the capsules. The reduction in capsule size allowed the graft to be confined to a specific graft site, enabling graft retrievability, reducing long-term complications and providing better long-term function than traditional alginate capsules. It is important to note that capsules implanted in the intraperitoneal space that cannot be removed pose safety concerns due to potential fibrotic reactions or adhesion to vital organs in the abdominal cavity (52). Edward A et al. added VEGF into polyethylene glycol maleimide (PEG-MAL) hydrogels, allowing on-demand VEGF release through enzymatic degradation. Rat islets encapsulated in PEG-MAL hydrogel secreted insulin during culture and were transplanted into the intestinal mesentery of healthy rats, and blood vessels were rebuilt within 4 weeks (53). In comparison with the encapsulation in conventional alginate-based materials, VEGF was released over an extended period of 7 days. Tissue samples were retrieved one week after implantation, and the macroscopic images clearly showed blood vessels extending from surrounding tissue to PEG-MAL hydrogel. Taken together, these results indicated that PEG-MAL could be used as islet transplantation vector and paved the way for the addition of other ECM components in PEG-MAL.

Nanoparticles (NPs) can be defined as particles which are 1–1000nm in size and possess colloidal properties. Enhance the delivery of attached or encapsulated substances by constructing nanoparticles with specific properties and release characteristics. Unlike other encapsulate methods that immobilize the cells or substances to be encapsulated in a micron-sized gel matrix, nanoencapsulation methods are usually based on the formation of nanomembranes around cells or organs (102, 103). Nanoencapsulation is a technology for encapsulating islets through conformal coating (104), mostly relying on the use of a nozzle method (105). As compared with conventional microcapsules, conformational coating allows for the formation of thin-films covering each individual islet (106). Both the size of the resulting materials and the thickness of the film are adjusted to the size and morphology of individual islets. This technology gives rise to nanocapsules (107, 108), for which the thickness of the protecting membrane favors the bi-directional diffusion of oxygen, nutrients and metabolites. Where possible, there is a correlation between the composition, physicochemical properties, frequency and route of nanoparticle implantation into the human body and tissue and blood toxicity, immunotoxicity and genotoxicity (109), and these factors cannot be ignored when designing nanoparticles.

Due to the small size of nanoparticles, they can cross cell membranes and enter blood and organs, and prolonged exposure of the body to these particles can lead to impaired clearance, inflammation and fibrosis (110). Also, the toxicity of the material affects the viability of the cells when the material is encapsuled around them. Toxicity can be categorized as acute (observed within less than 24 hours after single administration) or repeated (administration within 24 hours), subacute (observed within less than 1 month following repeated exposure), subchronic (observed within 1-3 months following repeated exposure) and chronic (observed after 3 or more months of chronic exposure) (109). Smaller nanoparticles have a higher specific surface area and cause larger contact region to cells which resulting in more toxic to cells. Nanoparticles are also easily removed by the body because their small size, which may run counter to our desire to keep encapsulated islets in the body for as long as possible playing their roles. Understanding the relationship between particle volume size and toxicity, clearance rate, degree of inflammatory fibrosis can help predict and design the appropriate size and frequency of implantation (111).

The usual shape of nanoparticles is spherical, which gives little attention to the effect of shape on nanocapsules. However, the shape of the nanoparticles affects the contact area of the material with the cells and the body internal environment, the diffusion distance of the material, the hydrodynamics in the body, etc. Different shapes of nanoparticles (mesoporous silica, long rod, short rod, spherical) were designed and it was found that rod-shaped nanospherical particles stay longer in the gastrointestinal tract than spherical ones. However, during excretion, spherical nanoparticles were cleared more quickly than rod-shaped nanoparticles (112). Therefore, the appropriate nanoparticle shape may be designed to achieve easier substance transport for therapeutic purposes.

In addition, nanoparticles obtain desirable surface chemistry through surface modifications, such as the introduction of PEG (103). Surface charge also affects the biosorption and half-life of nanoparticles (113). It is important to note that the above properties of nanoparticles may change upon entry into the human body (e.g., protein adsorption, etc.) and therefore, data on *in vivo* exposure are important for long-term safety assessment.

Several reports highlighted enhanced islet functionality under those conditions (114).

In clinical islet transplantations, the Edmonton protocol is the most standardized method (5). While the injection of non-encapsulated islets through the portal vein is appropriate, similar protocol applied to encapsulated cells may lead to severe complications, including the formation of blood clots due to the size of the system. In addition, the incomplete protection of the islets may trigger a strong immune response. Glucagon-like peptide-1 (GLP-1) stimulated insulin secretion in response to high glucose levels (54). Several studies pointed toward the reduction of the graft volume by encapsuling the islets within a biotin-PEG-GLP-1 conjugate using the layer-by-layer method, and the reduced size of the transplant was compatible with the Edmonton procedure without blocking the portal vein. Kim et al. developed cell-mimic polymersomes (PSomes) based on PEG-*b*-PLA (poly (ethylene glycol)-*b*-poly (DL-lactic acid)) for the coating neonatal porcine islet-like cell clusters (NPCCs). NHS-PSome-coated NPCCs and non-coated NPCCs were transplanted under the kidney capsule of C57/BL6 mice. The transplanted kidneys were removed after 14 days for immunohistochemical staining and it was found that there was significantly less infiltration of immune cells in the NHS-PSOME-coated group than in the uncoated NPCC group, without affecting the insulin secretion capacity of the coated cells (55). Neocrin Inc. transplanted PEG nano-encapsulated porcine islets under the renal capsule of diabetic rats. There was no significant fibrosis after 2 weeks and viable encapsulated porcine islet tissue existed more than 100 days after transplantation (56).

Despite these promising studies, the translation of islets loaded nanocapsules to the clinics is facing a series of limitations. The ultra-thin membranes may result in partial exposition of the coated islets to the immune system of the recipient, thus impairing their long-term survival. In addition, the retrieval of nanosized capsules remains challenging, which may trigger a series of potential adverse events (115, 116). While reinforcement of the thin-film membrane can be achieved by ultraviolet-light (UV-light) induced photo-crosslinking, such conditions may be detrimental to islet survival (117). Conformal coating, due to its tight attachment to islet cells, may cause the infiltration of the coating biomaterials into islets and their interaction with islets, leading to necrosis (118). Considering the above limitations, further work is needed to develop new methods for safer nanocapsule-based cell therapies (119). For example, the core-shell encapsulation technique has been developed, and several studies have shown that the use of core-shell encapsulation method is beneficial and can improve the cell survival rate by regulating the core materials (120, 121). The islets also have less chance of protruding from the side of the capsule, reducing the adverse immune response (68).

**Figure 5:**
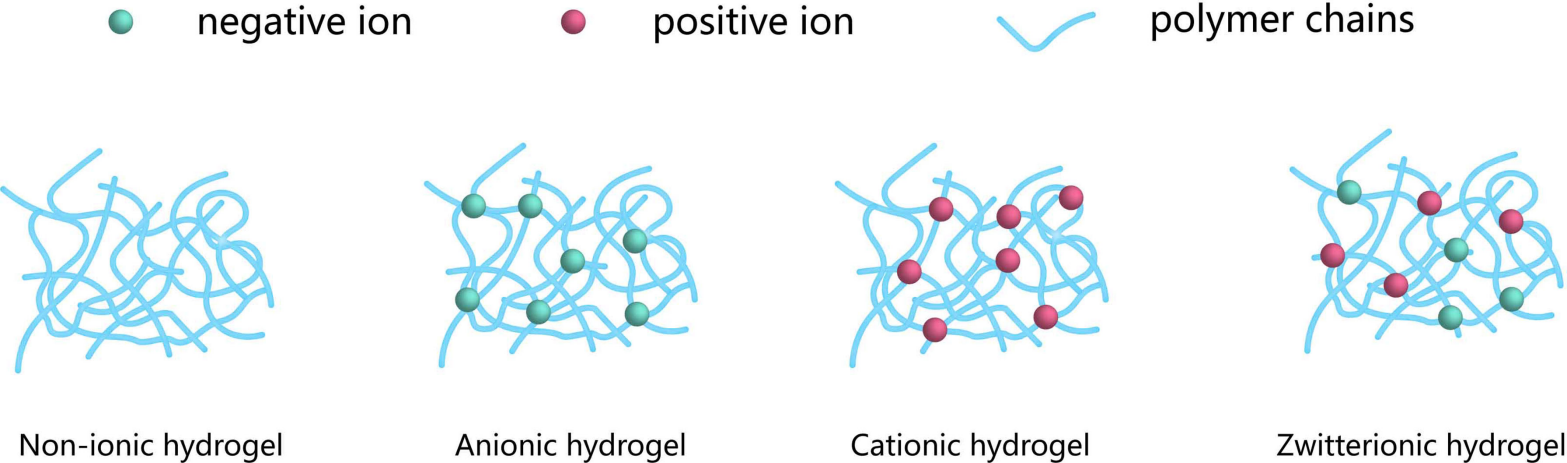
Classification of the encapsulation, according to the network electrical charge. Hydrogels may be categorized into three groups: Nonionic, Ionic (including anionic or cationic) and Zwitterionic. The blue networks are hydrogel polymers, the green spheres are negative ions, and the red spheres are positive ions.

Materials that mimic biological tissues primarily require excellent mechanical properties and long-term resistance to the formation of fibrosis caused by foreign body reactions. However, these standards are often difficult to achieve within a single material as they would require the combination of both hydrophobic and hydrophilic domains. Zwitterionic hydrogels are particularly promising for solving this problem (**Figure 5**) (122) and were applied to many medical fields. However, existing zwitterionic hydrogels do not have the long-term mechanical stability and antifouling performance needed for cell transplantation applications. Several strategies were developed to overcome the limitation of conventional zwitterionic hydrogels while maintaining their beneficial properties. Liu et al. (57). produced a poly (quaternized triazole carboxybetaine acrylamide) hydrogel which demonstrated reinforced mechanical properties and low non-specific protein adsorption. This material was investigated for the encapsulation of rat islets and further subcutaneous transplantation in immunocompetent diabetic mice. While mice treated with alginate-based microcapsules loaded with similar islet contents became gradually diabetic from day 18 after transplantation, these triazole containing zwitterionic hydrogels triazole moieties maintained normoglycemia for one month in 8 out of the 12 mice treated. In addition, post-retrieval histological analysis of the grafts showed abundant blood vessels and loose fibrotic overgrowth. Interestingly, the formation of blood vessels was significantly decreased for the mice who did not maintain normoglycemia.

## Combination of Natural and Artificial Hydrogels

Hydrogels resulting from natural polymers generally display favorable properties including angiogenesis, antibacterial activity and chemotaxis. Nevertheless, their quality is highly dependent on batch-to-batch variability and their mechanical properties hardly meet the required criteria for efficient islet transplantation. Therefore, the combination of natural and synthetic hydrogels offers the opportunity to correct the defects of natural components while maintaining their beneficial properties.

PLG is composed of copolymer of Lactide and glycolide (Poly (Lactide-co-glycolide). Besides controlling the distribution and density of transplanted islet cells in the scaffold, PLG’s dense pores are conducive to substance exchange and vascular reconstruction (123). T cells are the main immune cells responsible for T1D and islet transplantation rejection (124), and Fas ligand (FasL) can induce apoptosis by interacting with Fas on T cells to achieve immune tolerance (125). Co-transplantation of FasL protein overexpressed myoblasts with islets restored euglycemia without continuous immunosuppression (126). This approach has been applied to islet encapsulation. The researchers modified the surface of the pancreatic islets with FasL chimeric with streptavidin (SA-FasL) and combined the modified islets with a scaffold formed by coupling PLG and biotin, and transferred it into the epididymal fat pad of diabetic mice. The implanted islets showed sustained survival after short-term (15 days) immunosuppressive therapy, maintaining normal blood glucose for 200 days (59).

In addition to FasL modification of islet cells, co-embedding with regulatory T(Treg) cells is a novel approach to exploit immunosuppression. The ability of Treg cells to induce immune tolerance (127) provides a good idea for the treatment of autoimmune and alloimmune responses. PLG, as the scaffold for islet transplantation, co-located with Treg cells at the intra-abdominal fat of NOD mice, avoiding the instant blood-mediated inflammation caused by hepatic vein transplantation. This method induced long-term survival of transplanted cells without systemic immunosuppression, and realized the function of maintaining normal blood glucose (60). However, it leads to a non-specific inflammatory response, and the implantation process and biomaterials recruit antigen-presenting cell (APC) *in vivo*, inducing the secretion of inflammatory cytokines at the site of injury.

## Anti-Inflammatory Hydrogels

Current polymer hydrogel networks have been shown to block immune response cells and antibodies to protect islet cells, but permeation-selective barriers do not prevent low-molecular-weight cytotoxic molecules, such as interleukin-1β (IL-1β), tumor necrosis factor-α (TNF-α) from diffusing into the capsule material and damaging islet cells (35, 128). Approaches to encapsulate cells and tissues in anti-inflammatory hydrogels can solve this problem. Investigators have demonstrated that the natural polymeric hydrogels can exhibit strong anti-inflammatory activities, make them promising candidates as advanced therapeutics for tissue reconstruction applications.

Tannic acid (TA) is a polyphenolic natural product and an effective antioxidant (107). By using TA, antioxidants and neutral polymer poly(n-vinylpyrrolidone) (PVPON) multilayers to form a nano-thin encapsulation material PVPON/TA. After xenografting, PVPON/TA-encapsulated neonatal porcine islets were not defective in glucose responsiveness and had reduced expression of MHC-II and co-stimulatory molecules CD40, CD80 and CD86 in antigen-presenting cells (129).

Hyaluronic acid (HA) is the main component of the ECM, and the high molecular weight of HA has anti-inflammatory and immunosuppressive properties (130). *In situ* cross-linked hydrogels consisting of hydrazone-cross-linked aldehydes- and hydrazine-modified HAs are effective in preventing peritoneal adhesions in a rabbit side wall defect-cecal abrasion model (131). Cross-linking of dexamethasone modified HA to form an injectable hydrogel reduces TNF-α and IL-6 production by mouse primary macrophages and causes less inflammation compared to unmodified cross-linked HA (132). The Gel/Alg@ori/HA-PEI@siRNA-29a hydrogel prepared by Yang et al. on the basis of HA, by modifying HA, adding ori to shorten the inflammatory period and adding siRNA-29a to promote angiogenesis, was demonstrated in *in vivo* experiments to significantly promote diabetic wound healing and inhibit pro-inflammatory factors (IL-6 and TNF-α) (133).

Modification of artificial hydrogels with anti-inflammatory peptides and adhesion peptides is also a way to prepare anti-inflammatory hydrogels. Immobilization of peptides that inhibit cell-surface interleukin-1 (IL-1) receptors maintain the activity of cells encapsulated in PEGylated hydrogels exposed to a variety of cytokines including IL-1, TNF, and interferon (IFN). These peptide-modified hydrogels effectively protect encapsulated cells from β-cell-specific T cells and maintain insulin release from MIN6 cells (Mouse islet tumor cells) stimulated by glucose (58).

## Non-Hydrogels as Biomaterials for Cell Encapsulation

In addition to hydrogels, some non-hydrogels are also used as capsule materials, such as some biological cells and tissues, or some non-degradable synthetic polymer materials (**Table 2**).

**Table 2:** Non-hydrogels as biomaterials for cell encapsulation.

Material	Honor	Recipient	Graft	Site	Result	Reference
HEK293	Syrian hamsters	_	islets	_	no central necrosis and well glucose stimulation (5d)	(134)
Human amniotic membrane (HAM)	Human	Severe combined immunodeficiency mice	2,000 IEQ of human islets mixed with 0.4x 10^6^ HAECs	surface of the liver	all recipient attained euglycemia (by 2 wks)	(135)
NanoGland (Silicon)	Human	Nude mice	islets	subcutaneous	islet viability and responsiveness(120d)	(136)

In order to mitigate the adverse inflammatory response caused by the implantation of biomaterials, islet grafts were coated with leaving cells to improve their host biocompatibility. For instance, islet surface was covered with a biotinylated PEG-lipid layer and further conjugated with streptavidin-modified HEK293 cells (human endoderm kidney cell line) (134). The cell layer formed on the surface of the islets will be the immune barrier membrane. Such surface modification reduced the incidence of cell necrosis and maintained persistent glucose-stimulated insulin secretion ability of the protected islets.

Transplantation of islets to an extravascular site may not cause immediate inflammation, but on the other and, the revascularization process may also cause graft loss. Therefore, it is important to find the balance between angiogenesis and graft hypoxia. For these reasons, various human tissues have been combined with transplanted cells, such as the human amniotic membrane (HAM) and human amniotic epithelial cells (HAECs). Amniotic membrane consists of the epithelial layer and vascularless matrix offering an optimal scaffold for islet cells (135, 137). HAECs have a number of stem cell properties, such as potentially inducing angiogenesis (138) and being induced to differentiate into insulin-producing cells (139). Wanxing Cui et al. (135) placed decellularized 1.0x1.0cm^2^ HAM on the surface of the transverse membrane of the left lobe of the liver. Human islets and HAECs were mixed in Hank and the mixture was injected into the space between the HAM and the liver surface with a 200μL pipet tip. By the third day after transplantation, four of the seven recipient diabetic mice had normalized the blood glucose, and after two weeks seven recipient mice (100%) became normoglycemic.

A novel islet encapsulation silicon device, “NanoGland”, consists of an outer membrane with parallel nanochannels (3.6–40 nm) and perpendicular microchannels (20–60 microns) surrounding islets. The nanochannels are designed to provide immunoprotection and the microchannels are thought to enhance the engraftment. In addition to maintaining its own flexibility, the material still has precise nanoscale pores. Mice transplanted with allogeneic islets encapsulated whit the device showed function for up to 90 days and subcutaneous implantation of the NanoGland with human islets in mice showed survival of implants for more than 120 days. Analysis of the tissue surrounding NanoGland showed the presence of blood vessels, as well as the typical signs of fibrosis (136).

## Conclusions and Perspectives

The current methods for cell encapsulation include various approaches, such as nano-, micro-, macroencapsulation and numerous natural, bio-inspired and synthetic polymers have been tested. Although significant progress has been achieved and some preclinical trials have been performed, important hurdles still remain.

This article focuses on islet encapsulation, either by co-encapsulation or modification of the encapsulated material, in order to reduce the attack of the immune system on the graft and to maintain the cellular activity and physiological function as much as possible. Due to various constraints, islet capsule transplantation has also developed an increasing number of contents as well as material modifiers. For example, due to the shortage of donors, attention has been turned to xenotransplantation, such as exploring the transplantation of neonatal porcine islets. Neonatal porcine islets need to be cultured *in vitro* for four weeks to reach the same level of insulin secretion as adult islets (140). However, compared to mature porcine islets, neonatal islets are more likely to build up immune tolerance in the recipient, making them less susceptible to immune attack after maturation.

Therefore, the design of therapeutic hydrogels was envisioned. Currently this multifunctional hydrogel has been applied to the biomedical field, most commonly in multi-stage drug delivery (141), which is a new promising direction for future cellular encapsulation. The functional hydrogel assembles insulin and neonatal porcine islets into a system. After implantation into human body, the insulin in the early system is released slowly until the porcine islets mature. At later stage, hydrogel protects islets from immune attack. Free passage of nutrients and metabolites through the envelope, the mature pig islets secrete insulin at a later stage to achieve the capsule function. This multifunctional programmed hydrogel can be applied to various cells such as differentiating stem cells, etc., opening up a new avenue for the treatment of T1D.

The major limitations for large clinical application include the great variability of biomaterials, with insufficient biocompatibility leading to some degree of foreign body reaction and progressive fibrotic reactions. Based on previous studies that generally used one or two combined strategies to protect islet graft function, a multifunctional encapsulated hydrogel model with multiple functions is the way forward for our development.

With the continuous progress of technology, additional modifications of polymers should achieve higher degree of biological compatibility

## Author Contributions

LB and YW conceived and designed the review. QZ, CG-G, and YL wrote the manuscript. QZ and ZG prepared the figures. LB, YW, and SG-L reviewed and edited the manuscript. All authors read and approved the final manuscript.

## Funding

This study was sponsored by the National Science Foundation of China (81802504), the grant from Sichuan Medical Association (Q19037) and the grant from Chengdu Science and Technology Bureau (2021-YF05-00225-SN) for YW. YW and LB are supported by the International Innovation Cooperation Project of Sichuan Science and Technology Bureau (No.2022YFH0005) for islet xenotransplantation study. LB is supported by the Insuleman foundation, Child foundation and the foundation la Colombe.

## Conflict of Interest

The authors declare that the research was conducted in the absence of any commercial or financial relationships that could be construed as a potential conflict of interest.

## Publisher’s Note

All claims expressed in this article are solely those of the authors and do not necessarily represent those of their affiliated organizations, or those of the publisher, the editors and the reviewers. Any product that may be evaluated in this article, or claim that may be made by its manufacturer, is not guaranteed or endorsed by the publisher.
